# Waves of COVID-19 Pandemic: Effect on Ocular Surface Services at a Tertiary Eye Center in India

**DOI:** 10.7759/cureus.20719

**Published:** 2021-12-26

**Authors:** Anthony Vipin Das, Pragnya Rao, Swapna Shanbhag, Swati Singh, Sayan Basu

**Affiliations:** 1 Department of eyeSmart Electronic Medical Record (EMR) and AEye, L V Prasad Eye Institute, Hyderabad, IND; 2 Indian Health Outcomes, Public Health and Economics Research Center, L V Prasad Eye Institute, Hyderabad, IND; 3 Cornea and Anterior Segment Services, L V Prasad Eye Institute, Hyderabad, IND; 4 Center for Ocular Regeneration, L V Prasad Eye Institute, Hyderabad, IND

**Keywords:** emr, ocular surface disorders, big data, india, covid-19 pandemic

## Abstract

Objective

To describe the impact of lockdown and unlock phases of the coronavirus disease 2019 (COVID-19) pandemic on the ocular surface services at a tertiary eye care center in India.

Methods

This cross-sectional hospital-based study included 18,127 patients presenting between March 25, 2017, and July 31, 2021. A comparative analysis of the data was performed on the patients diagnosed with ocular surface disorders (allergic eye disease, cicatrizing conjunctivitis, dry eye, limbal stem cell deficiency, and ocular surface burns) and ocular surface surgeries (amniotic membrane grafting, keratoprosthesis, mucous membrane grafting, and simple limbal epithelial transplantation) presenting during the lockdown and unlock phases and the previous three years before COVID-19.

Results

The outpatient numbers dropped to 18.6% (172/954) and surgeries performed decreased to 2.8% (13/461) of pre-COVID-19 volumes during the lockdown phase. This was mainly because of a 74% reduction in the proportion of patients requiring inter-state travel to this tertiary care referral center. There was a gradual recovery of the outpatient numbers to 70.8% (565/798) and surgeries performed to 85.8% (109/127) of pre-COVID-19 volumes by February-March 2021. This gradual incremental trend was seen across all diagnoses and surgeries except for ocular burns, which showed an initial spike in the month of May followed by another sharp increase by the month of September that coincided with the gradual ease of lockdown regulations. The proportion of patients requiring inter-state travel showed an incomplete recovery to 77.2% of pre-COVID-19 distribution by March 2021.

Conclusion

The first year of the COVID-19 pandemic saw a drastic reduction in the outpatient numbers and surgical volume in the lockdown phase, which gradually recovered during the unlock period. However, the impact of the second wave was significant and is showing a gradual recovery in patients accessing eye care services.

## Introduction

“It was the best of times, it was the worst of times, it was the age of wisdom, it was the age of foolishness, it was the epoch of belief, it was the epoch of incredulity, it was the season of light, it was the season of darkness, it was the spring of hope, it was the winter of despair,” wrote Charles Dickens in the Tale of Two Cities, which has profound relevance in the current coronavirus disease 2019 (COVID-19) pandemic that is continuing to claim countless lives [1]. Patients had to overcome significant new challenges in the access to healthcare services across the country with the enforcement of the national lockdown in India last year [2]. The lockdown phase resulted in a drastic reduction in the outpatient numbers, and corneal disorders and trauma were the most common causes of patients seeking eye care services as reported from studies from India [3]. Rathi et al. reported equitable access to eye care services by gender, and the highest reduction of patient footfalls was seen in urban centers during the unlock 1.0 phase in India [4]. There is a need to identify vulnerable age groups who might not be able to access eye care services to identify health inequity, understand modifiable risk factors, and promote access with the use of technology tools such as telemedicine to address all those in need during these challenging times [5]. Ophthalmology practices need to address the challenges unique to the COVID-19 pandemic and be prepared to adapt to the changing practice patterns and the waxing and waning of the patient populations influenced by the evolving guidelines and regulations. Patients affected with ocular surface diseases have a prolonged chronic course of treatment that necessitates regular follow up and the sudden progression of the disease may warrant urgent surgical interventions. With this background, the authors describe a comparative report on the effect of lockdown, unlock, and the second wave of the COVID-19 pandemic on the ocular surface services at a tertiary eye care center in India.

## Materials and methods

Study design, period, location, and approval

Patients presenting between March 25, 2017, and July 31, 2021, to a tertiary eye care center located in India were included in this cross-sectional observational hospital-based study [6]. The patient or the parents or guardians of the patient signed a standard consent form for electronic data sharing for research purposes at the time of registration. None of the identifiable parameters of the patient information were used for the analysis of the data. The study was approved by the Institutional Ethics Committee, L V Prasad Eye Institute (approval number: LEC BHR-R-04-21-634), and adhered to the Declaration of Helsinki. The clinical data of each patient who underwent a comprehensive ophthalmic examination were entered into a browser-based electronic medical records system (eyeSmart Electronic Medical Record (EMR)) using a standardized template by trained ophthalmic personnel and supervised by an ophthalmologist [7].

Data retrieval and processing

A total of 18,127 patients of all ages who were diagnosed with ocular surface diagnosis, including allergic eye disease, cicatrizing conjunctivitis, dry eye, limbal stem cell deficiency, and ocular surface burns, and 3,143 patients who underwent surgical interventions, including amniotic membrane grafting, keratoprosthesis, mucous membrane grafting, and simple limbal epithelial transplantation, at a tertiary eye care center during the study period were included in this study. The data of these patients were retrieved from the electronic medical record database and segregated in a single Excel sheet (Microsoft Excel 2019, Microsoft Corporation, Redmond, WA). Data on patient demographics, ocular diagnosis, and surgical intervention were used for analysis. The Excel sheet with the required data was then used for analysis using the appropriate statistical software. The study duration was divided into four categories: pre-COVID-19 (between March 25, 2017, and March 24, 2020), lockdown (phase 1-4; between March 25, 2021, and May 31, 2021), unlock (phase 1-10; between June 1, 2020, and March 31, 2021), and second wave (between April 1, 2021, and July 31, 2021) [8]. The geographic categorization of patients was performed in relation to their location of origin to the eye care center. The “intra-city” patients presented from the same location of the eye center, “intra-state” patients presented from outside the city but from the same state of the eye center, “inter-state” patients presented from outside the state, and “international” patients presented from outside India. The demographic distribution and clinical presentation of the patients in these four categories were used for comparative analysis.

Statistical analysis

Descriptive statistics using mean ± standard deviation and median with interquartile range (IQR) were used to elucidate the demographic and clinical data using Microsoft Excel.

## Results

Overall trend of outpatient services

**Figure 1:**
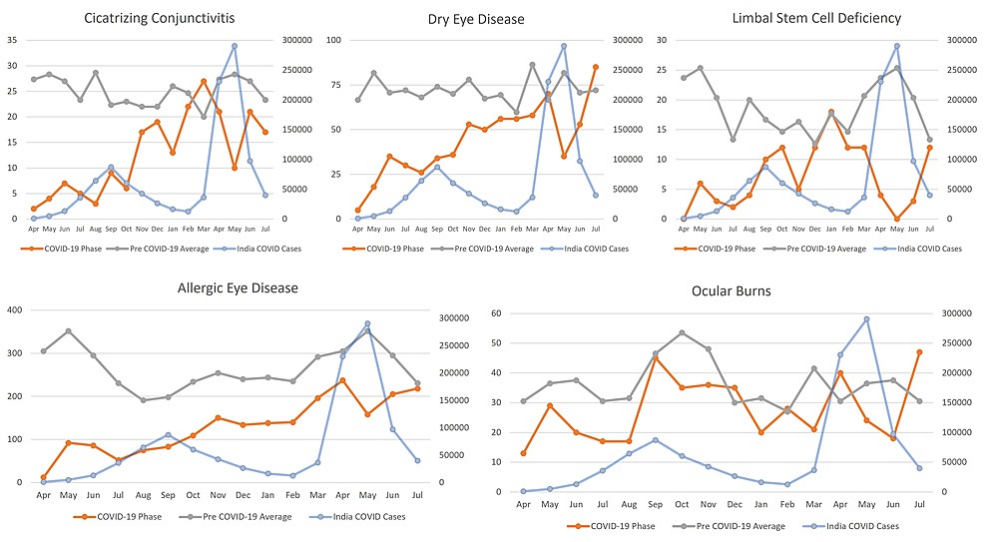
Distribution of allergic eye disease, cicatrizing conjunctivitis, dry eye, limbal stem cell deficiency, and ocular burns in patients presenting during the pre-COVID-19, lockdown (phase 1-4), unlock (phase 1-10), and second wave in India.

Overall, 18,127 patients diagnosed with ocular surface disorders (allergic eye disease, cicatrizing conjunctivitis, dry eye, limbal stem cell deficiency, and ocular burns) were presented during the study period. The number of patients seen during the lockdown phase with these diagnoses was significantly lower with an average of 2.77 (189/68) per day as compared to the pre-COVID-19 phase with an average of 12.97 (14,219/1,096) patients per day, and it increased to an average of 6.86 (2,081/303) during the unlock phase. There was no change in the mean age of the patients (28.65 ± 17.8 years vs. 28.23 ± 19.39 years) and median (26 (IQR: 14-41) years vs. 25 (11-42) years) during the COVID-19 phase (lockdown and unlock phases) as compared to the pre-COVID-19 phase. There was a slight decrease (30.48%) in the proportion of pediatric patients (≤16 years) during the COVID-19 phase as compared to the pre-COVID phase (36.62%). There was no major gender difference in access to care among the male (60.73% vs. 61.63%) and female (39.27% vs. 38.37%) patients. The trend of the ocular surface diagnosis over the three phases is detailed in Figure 1.

**Figure 2:**
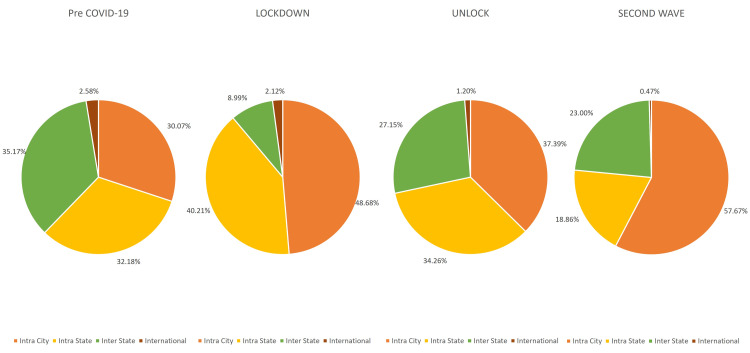
Distribution of outpatients with ocular surface disorders presenting during the pre-COVID-19, lockdown (phase 1-4), unlock (phase 1-10), and second wave in India.

With regards to the geographic presentation of patients, an increase was seen of 61.87% in intra-city and 24.98% in intra-state patients, and a proportional reduction of 74.43% was seen in patients requiring inter-state travel during the lockdown phase. There was an incomplete recovery in the proportion of outpatients to 77.19% for inter-state and 46.54% for international patients during the unlock phases. The detailed comparison of the geographic presentation in all three phases is described in Figure 2.

Specific trend of outpatient services

The yearly average of patients with ocular surface diseases reduced to 2,270 during the COVID-19 phase as compared to 4,740 during the pre-COVID-19 phase, while the monthly average also reduced from 395 to 189 patients. The yearly average of patients with allergic eye disease decreased from 3,069 to 1,267 patients, while the monthly average reduced from 256 to 106 patients. The yearly average of patients with cicatrizing conjunctivitis decreased from 295 to 134 patients, while the monthly average reduced from 25 to 11 patients and exceeded the pre-COVID-19 monthly average by March 2021. The yearly average of patients with dry eye disease decreased from 864 to 457 patients, while the monthly average reduced from 72 to 38 patients, which matched the pre-COVID-19 monthly average by February 2021. The yearly average of patients with limbal stem cell deficiency decreased from 216 to 96 patients, while the monthly average reduced from 18 to eight patients and showed a gradually increasing trend to match the pre-COVID-19 monthly average by October 2020. The yearly average of patients with ocular surface burns decreased from 445 to 316 patients, while the monthly average of ocular surface burns reduced from 37 to 26 patients and showed two distinct peaks to match the pre-COVID-19 monthly average in the months of May 2020 and September 2020. A comparative table of the patients presenting to the outpatient services is detailed in Table 1.

**Table 1:** Comparison of baseline characteristics during the pre-COVID, lockdown (phase 1-4), and unlocking (phase 1-10) period for patients with ocular surface disorders. * As compared to pre-COVID-19.

Variable		n	%	Pre-COVID-19	%	Lockdown (phase 1-4)	%	Unlock (phase 1-10)	%	p-value*
Outpatient cases		16,489	100	14,219	86	189	1	2,081	13	
Gender	Male	10,034	61	8,635	61	117	62	1,282	62	0.87
	Female	6,455	39	5,584	39	72	38	799	38	0.83
Age (years)	0-30	9,866	60	8,526	60	116	61	1,224	59	0.84
	31-40	2,199	13	1,848	13	36	19	315	15	0.04
	41-50	1,794	11	1,533	11	18	9	243	12	0.62
	51-60	1,424	9	1,222	8	13	7	189	9	0.44
	61-70	908	5	822	6	3	2	83	4	0.02
	71-100	298	2	268	2	3	2	27	1	0.77
Socio-economic status	Paying	14,244	86	12,195	86	170	90	1,879	90	0.65
	Nonpaying	2,245	14	2,024	14	19	10	202	10	0.15
Geographic status	Metropolitan	5,564	34	4,666	33	93	49	805	39	0.00
	Urban	5,550	34	4,889	34	57	30	604	29	0.39
	Rural	5,375	33	4,664	33	39	21	672	32	0.01
Distance to eye care center	Intra-city	5,146	31	4,276	30	92	49	778	37	0.00
	Intra-state	5,364	33	4,575	32	76	40	713	34	0.10
	Inter-state	5,583	34	5,001	35	17	9	565	27	<0.00001
Ocular surface disease	International	396	2	367	3	4	2	25	1	0.69
	Allergic eye disease	10,474	64	9,207	65	108	57	1,159	56	0.30
	Dry eye	3,048	18	2,591	18	25	13	432	21	0.13
	Ocular burns	1,205	7	889	6	43	23	273	13	<0.00001
	Cicatrizing conjunctivitis	1,018	6	884	6	6	3	128	6	0.10
	Limbal stem cell deficiency	744	5	648	5	7	4	89	4	0.59

Overall trend of surgical procedures

**Figure 3:**
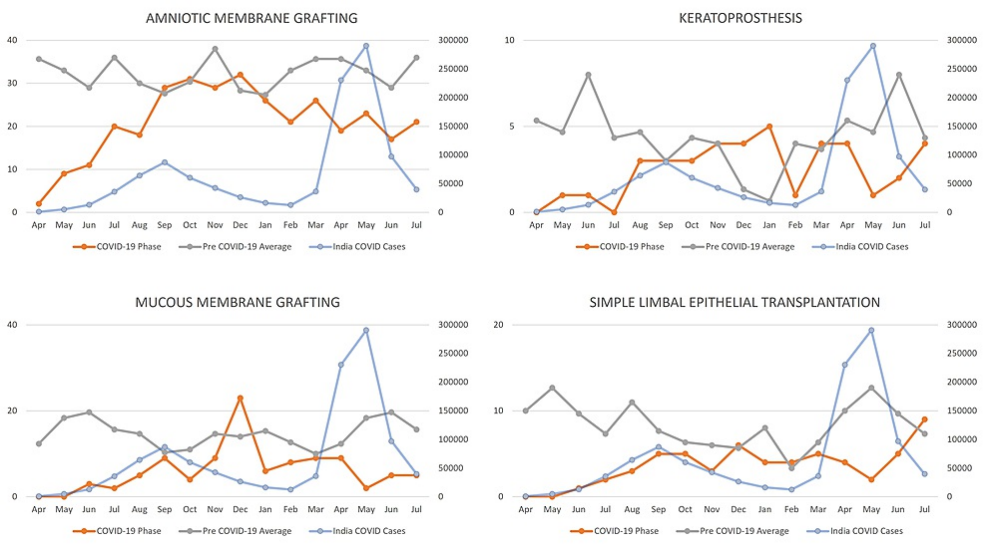
Distribution of amniotic membrane grafting, keratoprosthesis, mucous membrane grafting, and simple limbal epithelial transplantation surgeries during the pre-COVID-19, lockdown (phase 1-4), unlock (phase 1-10), and second wave in India.

During the study period, overall, 3,143 patients underwent the following surgical procedures: amniotic membrane grafting, keratoprosthesis, mucous membrane grafting, and simple limbal epithelial transplantation. The patients seen during the lockdown phase were significantly lower with a mean of 0.19 (13/68) per day as compared to the pre-COVID-19 phase with a mean of 2.27 (2,492/1,096) surgeries per day, which increased to a mean of 1.66 (506/303) during the unlock phase. There was no change in the mean age of the patients (36.45 ± 21.41 years vs. 37.13 ± 21.08 years) and median (36 (IQR:20-54) years vs. 35 (21-54) years) during the COVID-19 phase (lockdown and unlock phases) as compared to the pre-COVID-19 phase. There was no major difference in access to surgical care (20.42%) in the proportion of pediatric patients (≤16 years) during the COVID-19 phase as compared to the pre-COVID phase (19.22%). There was no major gender difference in access to care among the male (62.72% vs. 64.55%) and female (37.28% vs. 35.45%) patients. The trend of the ocular surface surgeries over the three phases is detailed in Figure 3.

**Figure 4:**
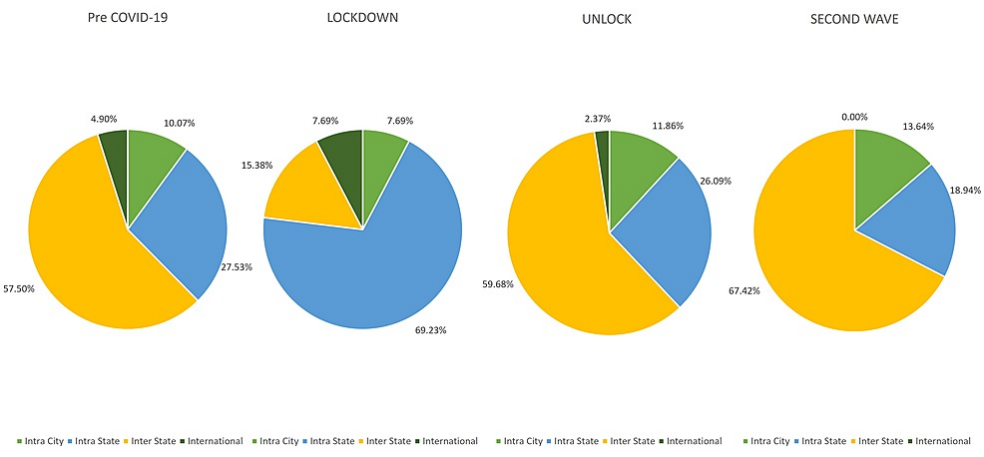
Distribution of ocular surface surgical interventions performed in patients presenting during the pre-COVID-19, lockdown (phase 1-4), unlock (phase 1-10), and second wave in India.

With regards to the geographic presentation of patients, an increase was seen of 251.49% in intra-state surgeries performed and a reduction of 26.75% was seen in surgeries for patients requiring inter-state travel during the lockdown phase. There was a near-complete recovery in surgeries performed (103.79%) for patients requiring inter-state travel and 117.73% for intra-city surgeries during the unlock phases. A detailed comparison of all three phases is described in Figure 4.

Specific trend of surgical procedures

The yearly average of patients with ocular surface surgeries reduced to 519 during the COVID-19 phase as compared to 831 during the pre-COVID-19 phase. The monthly average was also reduced to 43 surgeries (COVID-19 phase) from 69 surgeries (pre-COVID). The yearly average of amniotic membrane grafting decreased from 384 to 254 surgeries, while the monthly average reduced from 32 to 21 surgeries but showed a gradually increasing trend to match the pre-COVID-19 monthly average by September 2020. The yearly average of keratoprosthesis decreased from 48 to 29 surgeries, whereas the monthly average reduced from four to two surgeries and matched the pre-COVID-19 monthly average by September 2020. The yearly average of mucous membrane grafting decreased from 169 to 78 surgeries, while the monthly average reduced from 72 to 38 surgeries, which matched the pre-COVID-19 monthly average by September 2020. The yearly average of simple limbal epithelial transplants decreased from 94 to 38 surgeries, whereas the monthly average reduced from eight to three surgeries and showed a gradually increasing trend to match the pre-COVID-19 monthly average by December 2020. A comparative table of the patients undergoing ocular surface surgeries is detailed in Table 2.

**Table 2:** Comparison of baseline characteristics during the pre-COVID period, lockdown (phase 1-4), and unlocking (phase 1-10) for patients undergoing ocular surface surgeries. * As compared to pre-COVID-19.

Variable		n	%	Pre-COVID-19	%	Lockdown (phase 1-4)	%	Unlock (phase 1-10)	%	p-value*
Surgical cases		3,011	100	2,492	82	13	1	506	17	
Gender	Male	1,898	63	1,563	63	10	77	325	64	0.63
	Female	1,113	37	929	37	3	23	181	36	0.45
Age (years)	0-30	1,260	42	1,052	42	6	47	202	40	0.86
	31-40	471	15	383	15	2	15	86	17	0.996
	41-50	391	13	320	13	2	15	69	14	0.81
	51-60	382	13	312	13	1	8	69	14	0.64
	61-70	321	11	270	11	2	15	49	9	0.64
	71-100	186	6	155	6	0	0	31	6	NA
Socio-economic status	Paying	1,632	54	1,293	52	4	31	335	90	0.36
	Nonpaying	1,379	46	1,199	48	9	69	171	66	0.40
Geographic status	Metropolitan	434	15	359	14	1	8	74	15	0.54
	Urban	1,093	36	936	38	5	38	152	30	0.96
	Rural	1,484	49	1,197	48	7	54	280	55	0.81
Distance to eye care center	Intra-city	312	10	251	10	1	8	60	12	0.79
	Intra-state	827	28	686	28	9	69	132	26	0.03
	Inter-state	1,737	58	1,433	57	2	15	302	60	0.06
Ocular surface surgery	International	135	4	122	5	1	8	12	2	0.66
	Amniotic membrane grafting	1,406	47	1,152	46	11	85	243	48	0.14
	Keratoprosthesis	173	6	144	6	1	8	28	6	0.78
	Mucous membrane grafting	584	19	506	20	0	0	78	15	NA
	Simple limbal epithelial transplantation	320	11	282	11	0	0	38	8	NA
	Others	528	17	408	17	1	7	119	23	0.46

Second wave trends

Compared to the unlock phase with an average of 6.86 (2,081/303) outpatients per day, the number of patients with these diagnoses increased to an average of 10.47 (1,278/122) outpatients during the second wave phase. The outpatients decreased to 227 patients in the month of May 2021 and exceeded the pre-COVID average with 379 patients by July 2021. The surgeries from an average of 1.66 (506/303) during the unlock phase decreased to an average of 1.08 (132/122) surgeries during the second wave phase. The surgeries decreased to 28 surgeries in the month of May 2021 and never recovered compared to the pre-COVID average with 39 surgeries by July 2021. The monthly average of patients with allergic eye disease as compared to the pre-COVID phase average recovered to 218 patients by July 2021 and the monthly average of simple limbal epithelial transplantation surgery increased to nine surgeries by July 2021. With regards to geographic presentation, an increase of 54.23% was seen in outpatients from the intra-city level and an increase was seen of 12.98% in inter-state surgeries performed during the second wave phase as compared to the unlock phase.

## Discussion

This study sought to describe the impact of the lockdown and unlock phases of the COVID-19 pandemic on the ocular surface services at a tertiary eye care center in India. Our findings suggest that the outpatient numbers dropped to one-fifth and surgeries showed a sharp decline during the lockdown phase as compared to the pre-COVID-19 volumes. There was a three-fourth reduction in patients requiring inter-state travel. There was a gradual recovery seen in the outpatient numbers and surgeries performed as compared to the pre-COVID-19 volumes by February-March 2021. There was a distinct increasing trend seen with ocular surface burns during the initial unlock phases that coincided with the gradual ease of lockdown regulations by the government. There is still a challenge for patients requiring inter-state travel to access care due to the evolving travel restrictions due to the COVID-19 pandemic.

Our study has shown that allergic eye disease patients showed an increasing trend from April 2020 but never matched the monthly average of the pre-COVID-19 levels. This could be because of the significant reduction in outdoor activities due to staying indoors that might have caused a decreased risk of exposure to allergens. Patient volumes with cicatrizing conjunctivitis picked up by November 2020 as the travel restrictions eased and exceeded the monthly average of pre-COVID-19 volumes by March 2021 probably due to the backlog created due to the lockdown preventing access to care. Similarly, there was an increasing trend seen in patients with dry eye disease, which reached the pre-COVID-19 volumes by February 2021. Patients with limbal stem cell deficiency also showed a recovery by October 2020 indicating that the patients requiring chronic follow-up were able to access care. The patients presenting with ocular surface burns showed a distinct trend with two peaks in May 2020 followed by September 2020. This probably is because of the increased presentation of emergency cases during the lockdown phase and the second peak can be explained due to the gradual easing of the restrictions for industry and factories during the unlock phases. The Union Ministry of Home Affairs (MHA) had issued detailed guidelines under the Disaster Management Act, 2005 on restarting manufacturing industries after the lockdown period on May 1st, 2020 [9]. They cautioned against potential risk with hazardous chemicals and flammable materials to non-compliance with standard operating protocols (SOPs) during the lockdown. Improper enforcement of the safety codes and improperly labeled chemicals can pose serious health hazards to the individuals in these high-risk environments. The presentation of the ocular surface burns did not follow the expected peak seen during October that coincides with the festival season in India. The declining trend during this phase is probably because of the COVID-19 restrictions and also the governmental ban on firecrackers. The National Green Tribunal (NGT) of India issued a ban on the sale of firecrackers in a notice sent to 18 states and 122 non-attainment cities in November 2020 [10]. The Telangana High Court directed the state government to impose a ban on the sale and use of firecrackers owing to the prevailing COVID-19 situations and the risks to residents, especially senior citizens and children [11].

Our study also shows the increasing trend of amniotic membrane grafting surgeries performed peaking during September to October 2020 coinciding with the increasing trend of ocular surface burns. There was a steady recovery of keratoprosthesis by August 2020 due to the specialized nature of this surgery and the clearing of the backlog created by the lockdown phase. There was a steady recovery of mucous membrane grafting surgeries as well by September 2020 as patients continued to access care and the specialized nature of this procedure. Patients who required simple limbal epithelial transplantation steadily continued to access care and the surgical volume recovered by December 2020.

The geographic presentation of the patients showed a distinct pattern in the outpatient services across the three phases. There was a significant drop in the patients requiring inter-state travel during the lockdown phase due to the travel restrictions imposed by the government. The vacuum created by this was filled by the increase in both the intra-city and the intra-state patients during the unlock phase. On the other hand, with relation to the geographic pattern seen in surgical services, the unlock phase is a spitting image of the pre-COVID-19 phase with the exception of a decrease in international patients. There was a significant decrease in the patients requiring inter-state travel and an increase in the patients presenting from intra-state during the lockdown phase. However, it is good to see a significant number of patients have been able to travel and have access to surgical care during the unlock phase as compared to the pre-COVID-19 phase.

The second wave ravaged the country across its length and breadth and left us reeling with the staggering death tolls inflicted by the delta variant. The outpatients saw a decrease during the peak of the second wave in May 2021 and recovered to pre-COVID volumes by July 2021 and the surgical services showed a similar trend of decrease in volume by May 2021 but did not recover in numbers by July 2021. The sheer pace of the onslaught and progression of the second wave caused fear psychosis in the population that prevented travel unless considered an emergency, which was reflected in the increase in patients undertaking inter-state travel to access surgical care. As India begins to grapple with the aftermath of the second wave of COVID-19, we need to critically assess our experience of the first year to plan ahead. We need to identify the vulnerable groups such as pediatric age and patients requiring inter-state travel and use technology tools such as telemedicine to ensure continuity of care. There is a need for regular monitoring for the patients at risk especially as ocular surface diseases are chronic in nature, require prolonged follow-up, and have the risk of sudden worsening of the condition warranting urgent surgical intervention. We need to be cautious during the unlock phases as there is a risk posed to individuals returning to industrial work either due to non-compliance of SOPs in a high-risk environment or due to unskilled labor being employed due to the migrant crisis created due to the lockdown. With the rising second wave in India, it is imperative that we learn from our past experiences to prepare for an imminent lockdown that might come into effect with the exponential rise in active cases due to new variants of COVID-19.

## Conclusions

In conclusion, the authors present their experience on the impact of the COVID-19 pandemic on ocular surface services at a tertiary eye care center in India. The first year of the COVID-19 pandemic saw a drastic reduction in the outpatient numbers and surgical volume in the lockdown phase, which gradually recovered during the unlock period. However, the impact of the second wave was significant and is showing a gradual recovery in patients accessing eye care services.
